# Herpes Simplex Virus Type 2 Triggers Reactivation of Kaposi's Sarcoma-Associated Herpesvirus from Latency and Collaborates with HIV-1 Tat

**DOI:** 10.1371/journal.pone.0031652

**Published:** 2012-02-09

**Authors:** Qiao Tang, Di Qin, Zhigang Lv, Xiaolei Zhu, Xinting Ma, Qin Yan, Yi Zeng, Yuanyuan Guo, Ninghan Feng, Chun Lu

**Affiliations:** 1 State Key Laboratory of Reproductive Medicine, Nanjing Medical University, Nanjing, People's Republic of China; 2 Key Laboratory of Pathogen Biology of Jiangsu Province, Nanjing Medical University, Nanjing, People's Republic of China; 3 Department of Microbiology and Immunology, Nanjing Medical University, Nanjing, People's Republic of China; 4 Department of Clinical Laboratory, the Affiliated Nanjing First Hospital of Nanjing Medical University, Nanjing, People's Republic of China; 5 Department of Clinical Laboratory, Jiangsu Province Official Hospital, Nanjing, People's Republic of China; 6 Department of Microbiology and Immunology, Youjiang Medical College for Nationalities, Bose, People's Republic of China; 7 Department of Urology, the First Affiliated Hospital of Nanjing Medical University, Nanjing, People's Republic of China; National Institutes of Health, United States of America

## Abstract

Kaposi's sarcoma-associated herpesvirus (KSHV) infection was necessary but not sufficient for Kaposi's sarcoma (KS) development without other cofactors. Previously, we identified that both human immunodeficiency type 1 (HIV-1) Tat and herpes simplex virus 1 (HSV-1) were important cofactors reactivating KSHV from latency. Here, we further investigated the potential of herpes simplex virus 2 (HSV-2) to influence KSHV replication and examined the role of Tat in this procedure. We demonstrated that HSV-2 was a potentially important factor in the pathogenesis of KS, as determined by production of lytic phase mRNA transcripts, viral proteins and infectious viral particles in BCBL-1 cells. These results were further confirmed by an RNA interference experiment using small interfering RNA targeting KSHV Rta and a luciferase reporter assay testing Rta promoter-driven luciferase activity. Mechanistic studies showed that HSV-2 infection activated nuclear factor-kappa B (NF-κB) signaling pathway. Inhibition of NF-κB pathway enhanced HSV-2-mediated KSHV activation, whereas activation of NF-κB pathway suppressed KSHV replication in HSV-2-infected BCBL-1 cells. Additionally, ectopic expression of Tat enhanced HSV-2-induced KSHV replication. These novel findings suggest a role of HSV-2 in the pathogenesis of KS and provide the first laboratory evidence that Tat may participate HSV-2-mediated KSHV activation, implying the complicated pathogenesis of acquired immunodeficiency syndrome (AIDS)-related KS (AIDS-KS) patients.

## Introduction

Kaposi's sarcoma-associated herpesvirus (KSHV) was first detected in Kaposi's sarcoma (KS) tissues from a patient with acquired immunodeficiency syndrome (AIDS) by representational difference analysis [1]. The virus has been shown to correlate to all epidemiological forms of KS, primary effusion lymphoma (PEL) and a subset of multicentric Castleman's disease [2]–[5]. Like other herpesviruses, KSHV has two different phases in its life cycle, latency and lytic replication. Latency was characterized by persistence of the viral genome with expression of a limited set of viral genes. Once KSHV was reactivated from latency and entered the lytic cycle, most viral genes were expressed in an orderly fashion (immediate-early, early and late), leading to the production of infectious virions [6]–[8]. KSHV infection was necessary but not sufficient for KS development without other cofactors. We and others demonstrated that several agents, such as human immunodeficiency virus type 1 (HIV-1) transactivating protein Tat, herpes simplex virus type 1 (HSV-1), human herpesvirus 6 (HHV-6), human cytomegalovirus (HCMV) and HIV-1, have been proved to be cofactors reactivating KSHV from latency [9]–[13].

While sexually transmitted infections (STI) were associated with increased sexual transmission of HIV-1 and KS was the most common malignant tumor in patients with AIDS, more and more attentions were paid to the relationship of HIV-1, KSHV and the other sexually transmitted diseases (STD) pathogens [14]–[17]. A multi-center cross-sectional study in prisoners of Italian showed that, 20.7% prisoners had antibodies against KSHV, 21.2% prisoners had anti-HSV-2 antibodies, and 7.5% prisoners were HIV-1-positive. KSHV infection was associated with HSV-2 (P = 0.004) seropositivity. At multivariate analysis HSV-2-positivity was associated with HIV-1 (P<0.001) and KSHV infections (P = 0.003). The associations of KSHV and HSV-2 infection suggest sexual transmission of these viruses among Italian prison inmates [18]. In remote villages of the southwestern part of Papua New Guinea, the seropositivity of HSV-2 independently correlated with KSHV infection [19]. The research performed by A. Volpi et al in Northern Cameroon draw a similar conclusion [20]. These results suggest that HSV-2 infection was associated with sexual transmission of KSHV. HSV-2 could infect B cells and human vascular endothelial cells, the precursor of KS [21], [22]. Although HSV-2 and KSHV are not found in similar anatomic compartments during their latent infection, both reactivation and primary infection of HSV-2 occurred in patients, leading to appearance of HSV-2 viraemia [23]. Viraemia subsequently increased opportunities for HSV-2 to contact B and/or endothelial cells, which, maybe, previously had harbored the KSHV genome. Additionally, HIV-1 and KSHV do not generally infect the same cells, however, circulating Tat was secreted from infected cells and taken up by target cells [24], such as B and endothelial cells, which might also be latently infected by KSHV, resulting in changes in viral and cellular gene expression. These facts led us to hypothesize that HSV-2 may regulate KSHV replication and Tat plays a role in this procedure in KS or AIDS-KS patients.

To verify this hypothesis, in this study we performed kinetic studies of KSHV replication induced by HSV-2. We found that HSV-2 infection of PEL cell lines induced lytic replication of KSHV by activating Rta and inhibition of NF-κB pathway enhanced HSV-2-induced KSHV reactivation. Ectopic expression of Tat strengthened HSV-2-induced KSHV replication and Rta promoter activity. These novel findings are believed to be the first line of laboratory evidence that HSV-2 could influence KSHV replication and Tat exerted the enhancing function in this procedure.

## Results

### HSV-2 Infection of BCBL-1 Cells Results in Lytic Cycle Replication of KSHV

**Figure 1:**
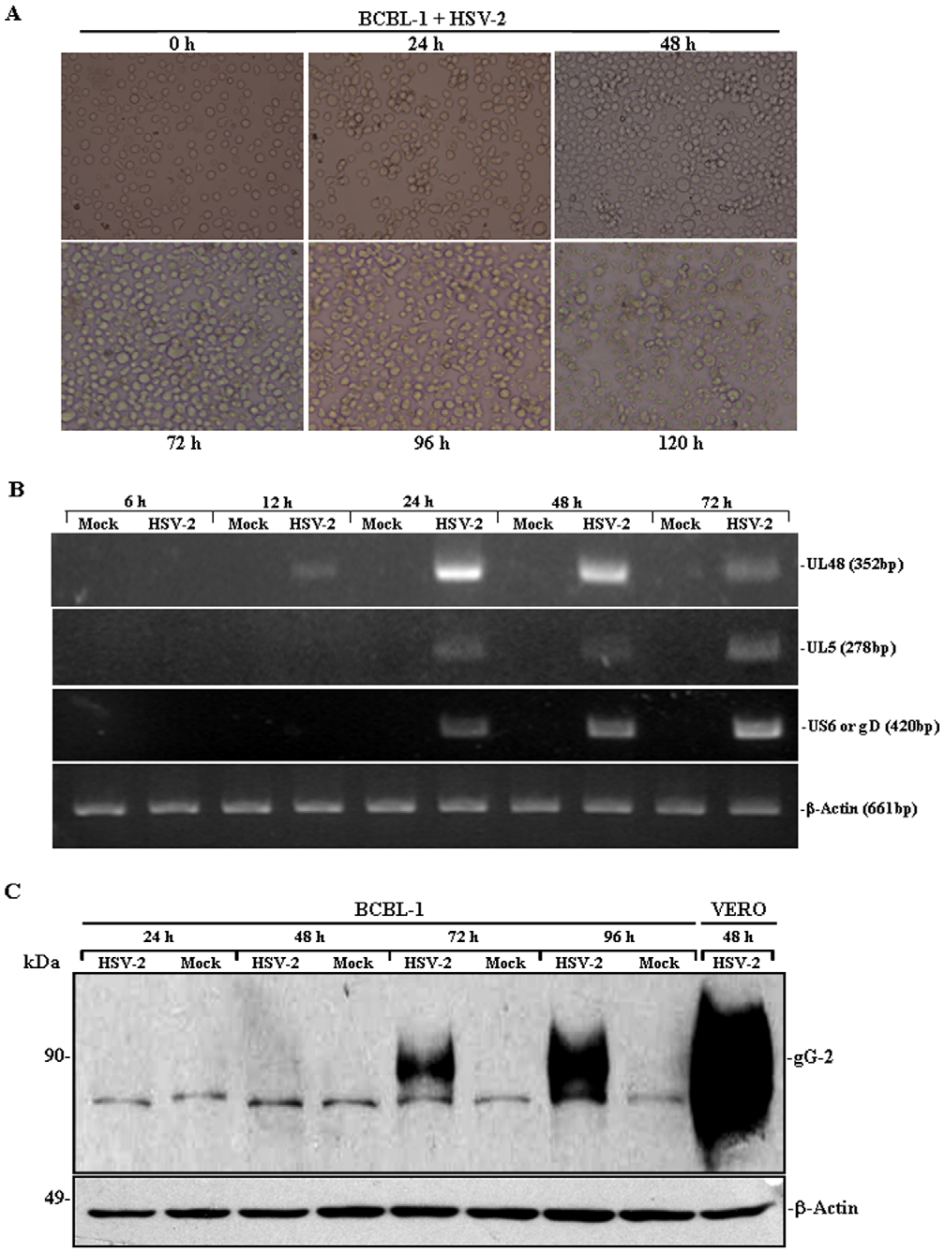
The susceptibility of BCBL-1 cells to HSV-2 infection. (**A**). **The CPE on BCBL-1 cells infected by HSV-2 (original magnification, ×40).** HSV-2 was inoculated into BCBL-1 cells for 0 to 120 hours and changes of cell morphology were observed under light microscope. (**B**). **RT-PCR analysis for HSV-2 immediate early, early, and late genes mRNA in HSV-2-infected BCBL-1 cells.** HSV-2 UL48, UL5 and US6 mRNA expression in BCBL-1 cells infected with Mock or HSV-2 for 6, 12, 24, 48, and 72 hours was detected by RT-PCR. β-Actin was readily detectable in all samples indicating the presence of amplifiable cDNA. (**C**). **Western blot analysis for HSV-2 structural protein expression in HSV-2-infected BCBL-1 cells.** BCBL-1 cells were infected with Mock or HSV-2 for 24, 48, 72 and 96 hours; or Vero cells were infected with HSV-2 for 48 hours (positive control). Whole cell lysate were subjected to Western blot with the indicated antibody. Results shown are from a representative experiment of three independent experiments with similar results.

To evaluate whether HSV-2 affected KSHV replication, we first determined the susceptibility of BCBL-1 cells to HSV-2. HSV-2 was inoculated into BCBL-1 cells and cell morphologic changes were observed by light microscope. As shown in Fig. 1A, BCBL-1 cells began to exhibit cytopathic effects (CPE) since 24 hours post-infection. With time going, the number of infected BCBL-1 cells showing the typical CPE, such as the formation of syncytia and polykaryocytes, gradually increased. Particularly, more than 80% of BCBL-1 cells exhibited the CPE at 120 hours post-inoculation (Fig. 1A) while only less than 5% of Mock-treated BCBL-1 cells showed CPE at this time point (data not shown). To determine whether HSV-2 could replicate in BCBL-1 cells, the mRNA expression of a HSV-2 immediate early (IE) gene, UL48, which encodes transactivating tegument protein VP16 [25], was detected by RT-PCR. We found that the mRNA of UL48 started to express in BCBL-1 cells since 12 hours post-infection (pi) and reached a peak at 24 hours pi, then decreased by degrees (Fig. 1B). Further, the experiment was designed to detect the UL5 gene, an early gene of HSV-2 encoding helicase-primase helicase subunit and a late gene, US6, which encodes viral envelope glycoprotein D (gD) [26], [27]. It was demonstrated that both UL5 and US6 mRNAs began to express at 24 hours pi and gradually increased with time going (Fig. 1B). Finally, HSV-2 structure protein gG2 (the type-specific IgG encoded by a late gene) [28] was detected by Western blot. We found that HSV-2-infected BCBL-1 cells started to express gG2 since 72 hours pi and continued to increase expression of gG2 at 96 hours (Fig. 1C). These data indicated that HSV-2 infected BCBL-1 cells and replicated.

**Figure 2:**
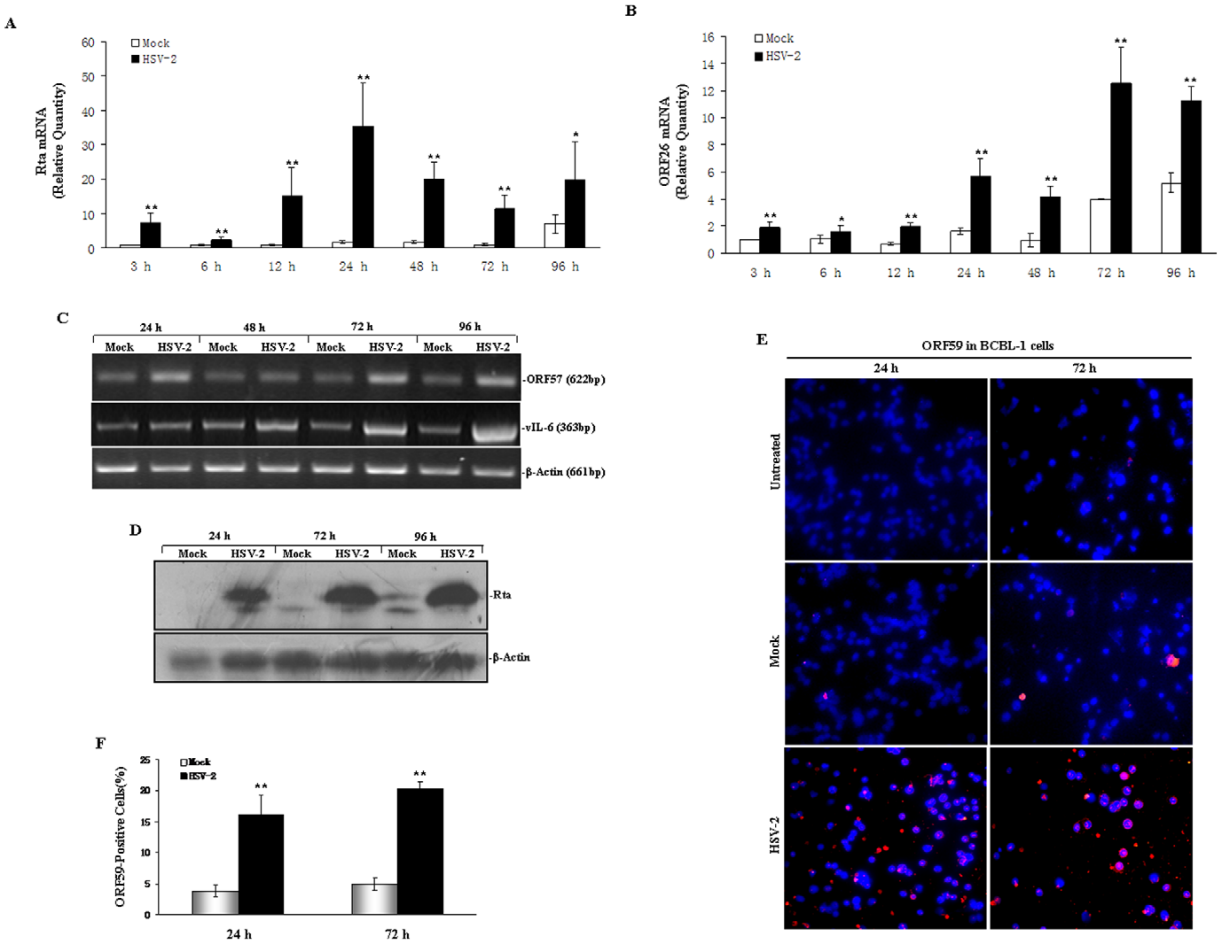
Expression of KSHV lytic cycle RNA and protein in HSV-2-infected BCBL-1 cells. (**A**). **Rta mRNA expression in HSV-2-infected BCBL-1 cells.** Rta mRNA expression in BCBL-1 cells infected with Mock or HSV-2 for 3, 6, 12, 24, 48, 72 and 96 hours was quantitated by RT-qPCR. Relative quantities of Rta expression represented on the y-axis. Results shown were from three independent experiments performed in triplicate. * *p*<0.05 and ** *p*<0.01 for Student's t-test versus mock group, respectively. (**B**). **ORF26 mRNA expression in HSV-2-infected BCBL-1 cells.** ORF26 mRNA expression in BCBL-1 cells infected with Mock or HSV-2 for 3, 6, 12, 24, 48, 72 and 96 hours was measured by RT-qPCR. Relative quantities of ORF26 expression represented on the y-axis. Results shown were from three independent experiments performed in triplicate. * *p*<0.05 and ** *p*<0.01 for Student's t-test versus mock group, respectively. (**C**). **RT-PCR analysis for ORF57 and vIL-6 mRNA in BCBL-1 cells infected with HSV-2.** ORF57 and vIL-6 mRNA expression in BCBL-1 cells infected with Mock or HSV-2 for 24, 48, 72 and 96 hours was detected by RT-PCR. β-Actin was readily detectable in all samples indicating the presence of amplifiable cDNA. (**D**). **Rta protein expression in BCBL-1 cells infected by HSV-2.** BCBL-1 cells were infected with HSV-2 or mock for 24, 72 and 96 h and whole-cell lysates were subjected to Western blot with Rta antibody. The membrane was stripped and reprobed with anti-actin antibody as a loading control. Results shown are from a representative experiment of three independent experiments with similar results. (**E**). **Immunofluorescence assay staining of BCBL-1 cells infected with HSV-2 (original magnification, 40×).** KSHV lytic protein ORF59 expression in normal BCBL-1 cells (untreated) and BCBL-1 cells infected with mock or HSV-2 for 24 and 72 h was detected by immunofluorescence assay staining with ORF59 MAb. (**F**). **Quantification of results in** (**E**). Quantitative and statistic methods were described in the text. ** *p*<0.01 for Student's t-test versus mock group.

To examine whether HSV-2 could activate KSHV replication, RT-qPCR was performed. Analysis of data from three independent experiments demonstrated that, on average, Rta (replication and transcription activator, Rta; the molecular switch gene of KSHV; also known as ORF50) [8], [29] mRNA expression in HSV-2-infected BCBL-1 cells increased 7.26-fold at 3 hours, 2.52-fold at 6 hours, 15.77-fold at 12 hours, 20.92-fold at 24 hours, 11.44-fold at 48 hours, 11.02-fold at 72 hours, and 2.81-fold at 96 hours, respectively, when compared to Mock-treated BCBL-1 cells (Fig. 2A). Meanwhile, ORF26 (viral minor capsid protein expressed only during lytic KSHV replication) [30] mRNA showed a similar expression pattern as Rta (Fig. 2B). To determine whether other KSHV lytic genes were also up-regulated in HSV-2-infected BCBL-1 cells, RT-PCR analysis was performed to detect the expression of ORF57 and vIL-6 transcripts. All mRNAs of these genes in HSV-2-infected BCBL-1 cells were increased from 24 to 96 hours when compared to their corresponding controls (Fig. 2C). These data suggest that HSV-2 might activate KSHV lytic cycle RNA replication. Next, we asked whether induction of KSHV lytic cycle RNA replication by HSV-2 also resulted in induction of lytic cycle proteins, the expressions of lytic cycle protein Rta and ORF59 were examined. As shown in Fig. 2D, for each of the time points (24, 72 and 96 hours), the level of Rta expression was increased in HSV-2-infected BCBL-1 cells compared to Mock-treated cells. Similarly, the expression of KSHV ORF59 protein was significantly increased in HSV-2-infected BCBL-1 cells at 24 and 72 hours compared with the corresponding control (Fig. 2E and 2F).

### HSV-2 Infection of BCBL-1 Cells Results in Release of Infectious Virions of KSHV

**Figure 3:**
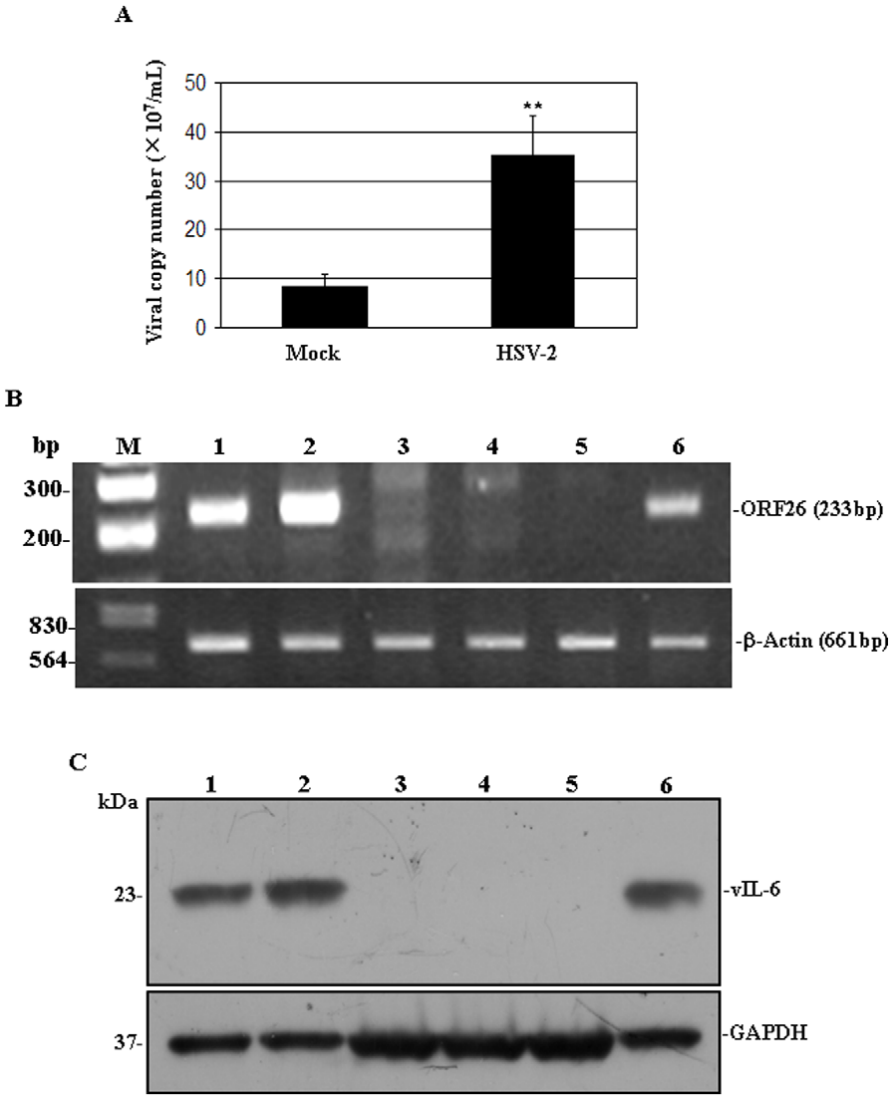
HSV-2 infection of BCBL-1 cells results in release of infectious virions of KSHV. (**A**). **Real-time DNA-PCR analysis for the viral copy number of KSHV.** BCBL-1 cells were infected with HSV-2 or Mock. At 72 hours postinfection, supernatant virus was collected, concentrated, and used for real-time DNA-PCR analysis. Results shown were from three independent experiments performed in triplicate. ** *p*<0.01 for Student's t-test versus mock group. (**B**). **RT-PCR analysis for ORF26 mRNA in BCBL-1 or HEK293 cells treated with supernatant from HSV-2-infected BCBL-1 cells.** ORF26 mRNA expression in BCBL-1 cells treated with TPA (**Lane 1**), HEK293 cells treated for 48 hours with supernatant from TPA-treated BCBL-1 cells for 72 hours (**Lane 2**), HEK293 cells cultured alone for 27 hours (**Lane 3**), HEK293 cells infected with HSV-2 for 27 hours (**Lane 4**), HEK293 cells treated for 27 hours with supernatant from Mock-treated BCBL-1 cells for 72 hours (**Lane 5**), and HEK293 cells treated for 27 hours with supernatant from HSV-2-infected BCBL-1 cells for 72 hours (**Lane 6**) were detected by RT-PCR. (**C**). **Western blot for detection of vIL-6 expression in BCBL-1 cells or HEK293 cells treated with supernatant from HSV-2-infected BCBL-1 cells.** vIL-6 expression in BCBL-1 cells treated with TPA (**Lane 1**), HEK293 cells treated for 48 hours with supernatant from TPA-treated BCBL-1 cells for 72 hours (**Lane 2**), HEK293 cells cultured alone for 27 hours (**Lane 3**), HEK293 cells infected with HSV-2 for 27 hours (**Lane 4**), HEK293 cells treated for 27 hours with supernatant from Mock-treated BCBL-1 cells for 72 hours (**Lane 5**), and HEK293 cells treated for 27 hours with supernatant from HSV-2-infected BCBL-1 cells for 72 hours (**Lane 6**) were detected by Western blot.

Theoretically, KSHV lytic cycle replication induced by HSV-2 would subsequently produce infectious progeny virions. To test this hypothesis, experiments were designed to detect KSHV virions in the supernatant from HSV-2-infected BCBL-1 cell cultures. The supernatant of HSV-2-infected BCBL-1 cells was collected and divided into two parts. The 1^st^ part was used for viral DNA extract. Purified viral DNA was used for real-time DNA-PCR analysis. As shown in Fig. 3A, KSHV viral copy number in HSV-2-infected BCBL-1 cells was increased 4.14-fold at 72 hours when compared to Mock-infected BCBL-1 cells. To further determine whether these viral particles were infectious, the 2^nd^ part of supernatant was employed to infect HEK293 cells, which were previously shown to be permissive cells for KSHV [31]. After 27 hours infection, the total RNA was isolated and RT-PCR was performed to amplify KSHV specific sequence, ORF26. The results demonstrated that ORF26 mRNA was readily expressed in HEK293 cells infected with the supernatant from HSV-2-infected BCBL-1 cells, but not in HEK293 cells treated with the supernatant from Mock-treated BCBL-1 cells (Fig. 3B). And vIL-6 protein was also detected in HEK293 cells infected with the supernatant from HSV-2-infected BCBL-1 cells by Western blot (Fig. 3C).

### HSV-2 Activates KSHV Lytic Cycle Replication possibly by Activating Rta

**Figure 4:**
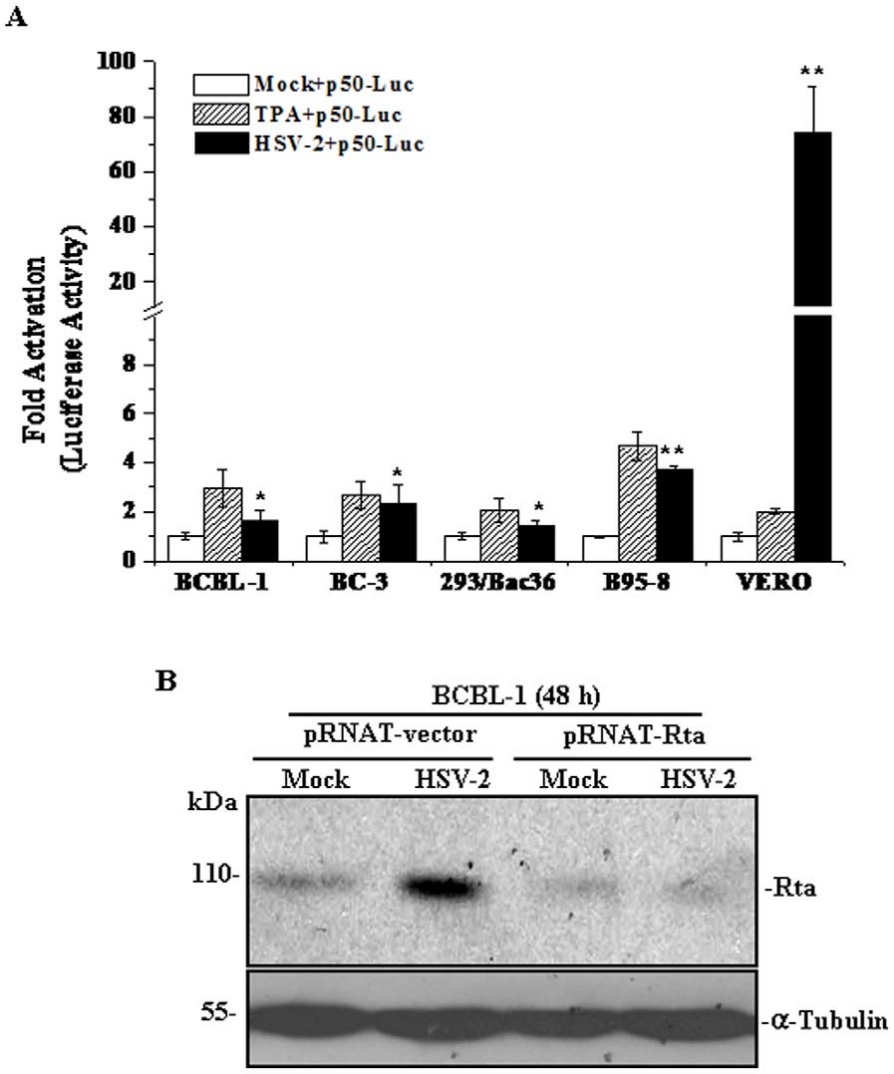
HSV-2 activates KSHV lytic cycle replication via Rta. (**A**). **HSV-2 infection promotes induction of KSHV Rta promoter activity.** BCBL-1, BC-3, 293/Bac36, B95-8 and Vero cells were infected with Mock (**Mock+p50-Luc**) or HSV-2 (**HSV-2+p50-Luc**), or treated with TPA (**TPA+p50-Luc**), and then cotransfected with the firefly luciferase reporter construct p50-Luc (0.4 µg) and a Renilla luciferase construct pRL-TK (0.02 µg). Luciferase activities were measured, normalized to inner control, and presented as fold increase (*n*-fold). All data points were the averages of three independent experiments performed in triplicate. * *p*<0.05 and ** *p*<0.01 for Student's t-test versus Mock+p50-Luc group, respectively. (**B**). **Western blot analysis for Rta protein in si-construct-transfected BCBL-1 cells infected with HSV-2.** BCBL-1 cells were transfected with pRNAT-Rta or pRNAT-vector, and then infected with HSV-2 or Mock for 48 hours. Whole cell lysate were subjected to Western blot with the indicated antibody.

To explore whether HSV-2 induced KSHV replication by activating Rta, we examined the effect of HSV-2 infection on Rta promoter activity in several cell lines. Cells transfected with p50-Luc demonstrated enhanced levels of luciferase expression (3.75, 4.92 and 2.13-fold increase in BCBL-1, B95-8 and Vero cells, respectively) by treatment with TPA (used as a positive control). Notably, HSV-2 infection resulted in a significant increase in luciferase expression [1.83, 3.87, and 76.33-fold increase in BCBL-1 (*P* = 0.039), B95-8 (*P* = 0.001) and Vero (*P* = 0.002) cells, respectively] compared to the corresponding control (Fig. 4A). To confirm this result, luciferase assays were also performed in the other KSHV-positive cell lines, such as BC-3 (a type of PEL) and 293/Bac36. It was demonstrated that HSV-2 infection lead to a 2.31- and 1.44-fold increase in luciferase expression in BC-3 (*P* = 0.047) and 293/Bac36 (*P* = 0.032) cells, respectively, when compared to the corresponding control (Fig. 4A). To extend these results, a siRNA construct, which was previously designed to silence KSHV Rta [11], was introduced into HSV-2-infected BCBL-1 cells. As shown in Fig. 4B, Rta protein in HSV-2-infected BCBL-1 cells further transfected with pRNAT-Rta was markedly decreased at 48 hours when compared with the corresponding controls. These observations collectively suggest that HSV-2 activates KSHV replication possibly by activating Rta.

### Inhibition of NF-κB Signaling Enhances HSV-2-Induced KSHV Replication

**Figure 5:**
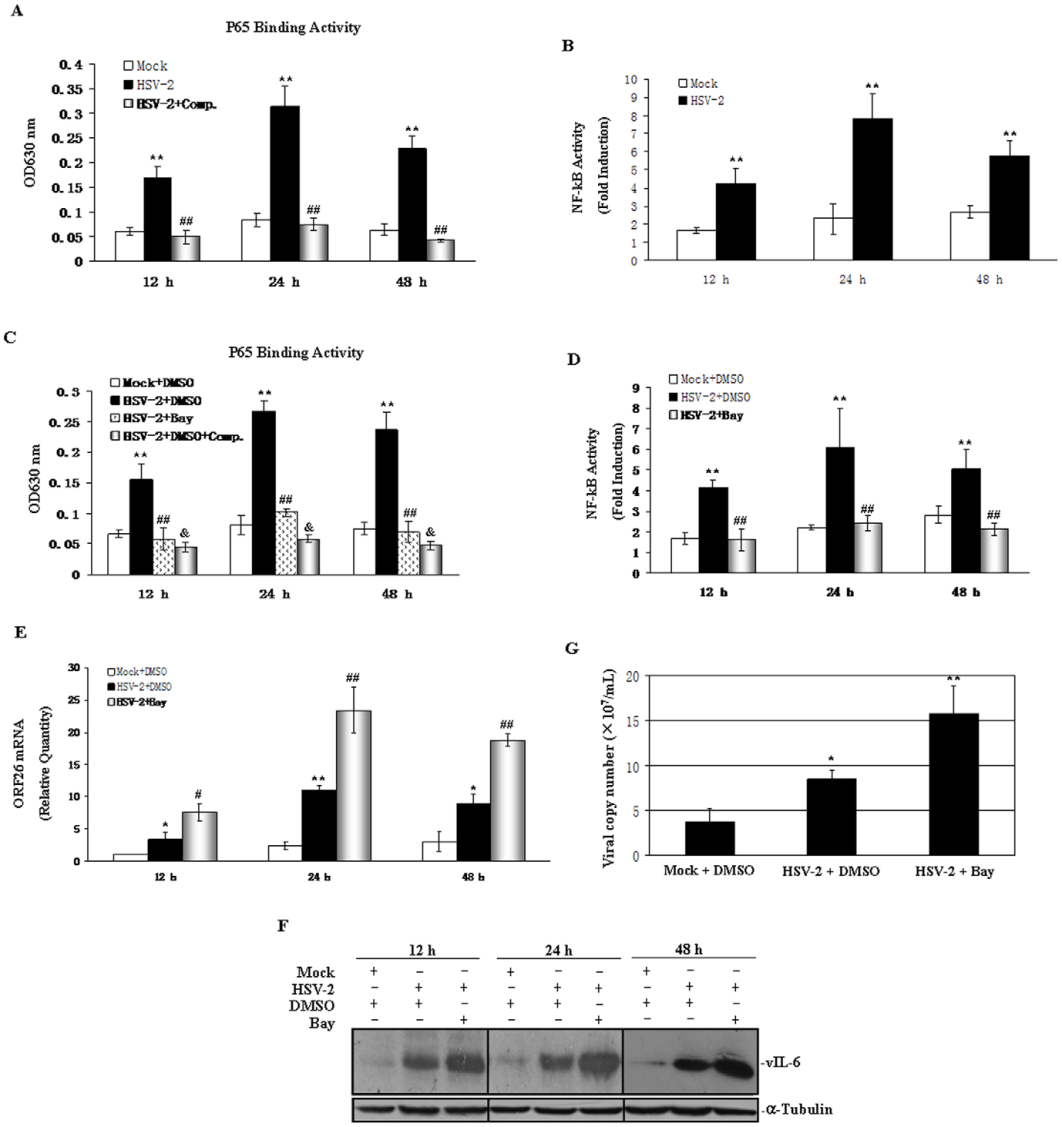
Inhibition of NF-κB signaling enhances HSV-2-induced KSHV replication. (**A**). **HSV-2 infection elevated the P65 level of NF-κB pathway in BCBL-1 cells.** BCBL-1 cells were infected by HSV-2 or Mock for 12, 24 and 48 hours. Then cells were collected and nuclear proteins were extracted for ELISA-based NF-κB activity assay. Excess competitor oligonucleotides were coincubated with the nuclear protein of HSV-2-infected BCBL-1 cells for competition assays (HSV-2+Comp.). Results presented were the statistic of three independent experiments performed in triplicate. ** *p*<0.01 and ^##^ *p*<0.01 for Student's t-test versus Mock and HSV-2 groups, respectively. (**B**). **HSV-2 infection increased NF-κB activity in BCBL-1 cells.** BCBL-1 cells adsorbed with HSV-2 or Mock for 1 hour, then were transfected with an NF-κB-dependent firefly luciferase reporter construct (0.4 µg) together with a Renilla luciferase construct pRL-TK (0.02 µg) for 12, 24 and 48 hours. Whole-cell extracts were prepared, and the firefly as well as the Renilla luciferase activity was measured. The experiment was performed three times in parallel, and the mean ± s.e.m is shown. ** *p*<0.01 for Student's t-test versus Mock group. (**C**). **A pharmacologic inhibitor of IKK–NF-κB activation, Bay 11-7082, inhibited the binding activity of P65 of NF-κB pathway in HSV-2-infected BCBL-1 cells.** BCBL-1 cells were pre-treated by Bay 11-7082 (5 µM) or DMSO for 1 hour followed by HSV-2 or Mock infection for 12, 24 and 48 hours. Then cells were collected and nuclear proteins were extracted for ELISA-based NF-κB activity assay. Excess competitor oligonucleotides were coincubated with the nuclear protein of DMSO-pre-treated and HSV-2-infected BCBL-1 cells for competition assays (HSV-2+DMSO+Comp.). Results presented were the statistic of three independent experiments performed in triplicate. ** *p*<0.01 for Student's t-test versus Mock+DMSO group; ^##^ *p*<0.01 and ^&^ *p*<0.01 for Student's t-test versus HSV-2+DMSO group, respectively. (**D**). **Bay 11-7082 inhibited HSV-2-induced activity of NF-κB in BCBL-1 cells.** BCBL-1 cells were pre-treated by Bay 11-7082 (5 µM) or DMSO for 1 hour followed by HSV-2 or Mock infection for 1 hour and then were transfected with an NF-κB-dependent firefly luciferase reporter construct (0.4 µg) together with a Renilla luciferase construct pRL-TK (0.02 µg) for 12, 24 and 48 hours. Whole-cell extracts were prepared, and the firefly as well as the Renilla luciferase activity was measured. The experiment was performed three times in parallel, and the mean ± s.e.m is shown. ** *p*<0.01 and ^##^ *p*<0.01 for Student's t-test versus Mock+DMSO and HSV-2+DMSO groups, respectively. (**E**). **Bay 11-7082 enhanced ORF26 mRNA expression in HSV-2-infected BCBL-1 cells.** BCBL-1 cells were pre-treated by Bay 11-7082 (5 µM) or DMSO for 1 hour followed by HSV-2 or Mock infection for 12, 24 and 48 hours. ORF26 mRNA expression in BCBL-1 cells was measured by RT-qPCR. Relative quantities of ORF26 expression represented on the y-axis. Results shown were from three independent experiments performed in triplicate. * *p*<0.05 and ** *p*<0.01 for Student's t-test versus Mock+DMSO group, respectively; ^#^ *p*<0.05 and ^##^ *p*<0.01 for Student's t-test versus HSV-2+DMSO group, respectively. (**F**). **Bay 11-7082 enhanced vIL-6 expression in HSV-2-infected BCBL-1 cells.** BCBL-1 cells were pre-treated by Bay 11-7082 (5 µM) or DMSO for 1 hour followed by HSV-2 or Mock infection for 12, 24 and 48 hours. Whole cell lysate were subjected to Western blot with the indicated antibody. (**G**). **Real-time DNA-PCR analysis for the viral copy number of KSHV.** BCBL-1 cells were pre-treated by Bay 11-7082 (5 µM) or DMSO for 1 hour followed by HSV-2 or Mock infection. At 24 and 48 hours postinfection, supernatant virus was collected, concentrated, and used for real-time DNA-PCR analysis. Results shown were from three independent experiments performed in triplicate. * *p*<0.05 and ** *p*<0.01 for Student's t-test versus Mock+DMSO and HSV-2+DMSO groups, respectively.

**Figure 6:**
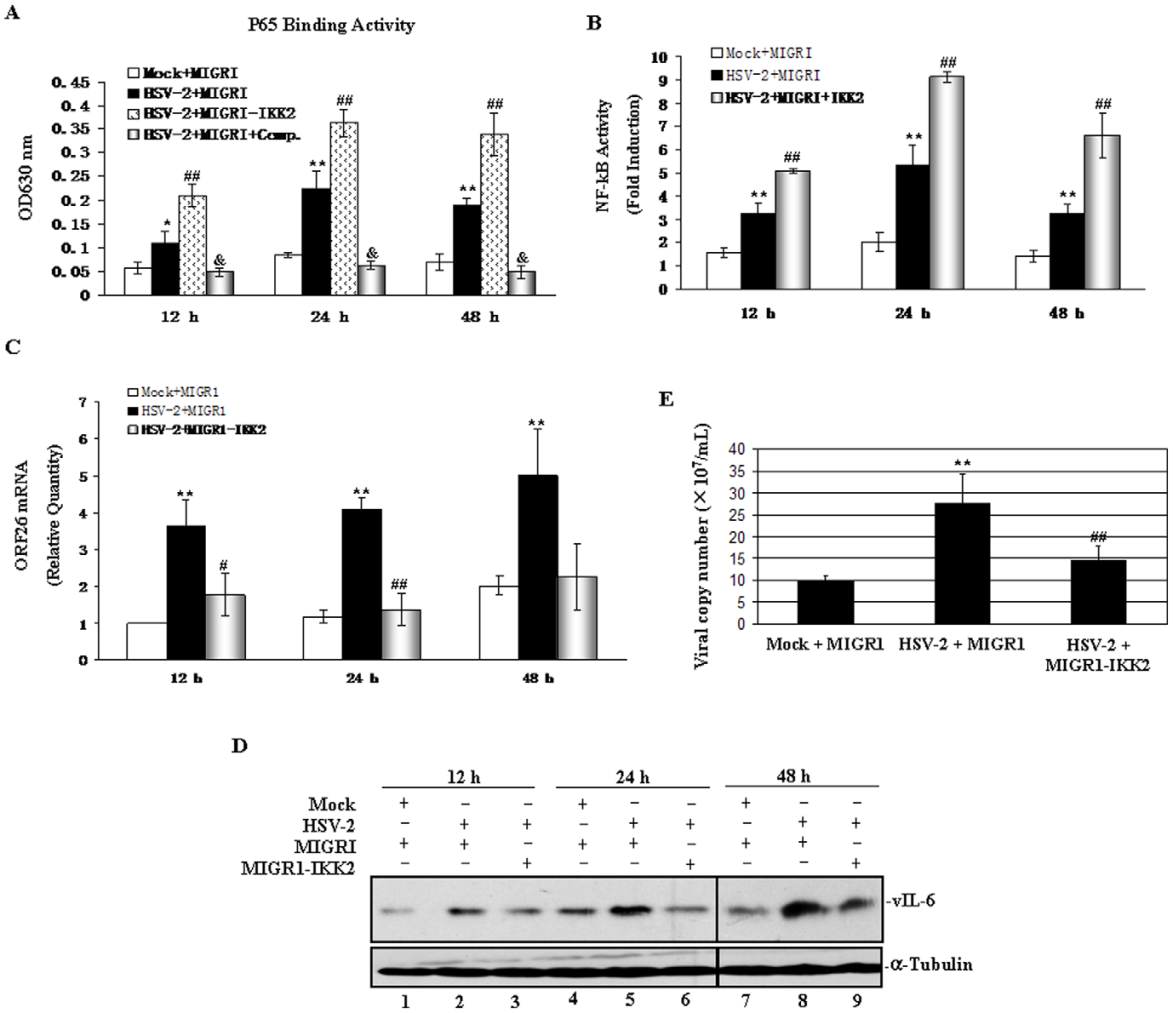
Elevating NF-κB signaling inhibits HSV-2-induced KSHV replication. (**A**). **Transfection of IKK_2_EE promoted the binding activity of P65 of NF-κB pathway in HSV-2-infected BCBL-1 cells.** BCBL-1 cells were infected by HSV-2 or Mock for 1 hour, and then were transfected by IKK_2_EE or MIGRI for 12, 24 and 48 hours. Cells were collected and nuclear proteins were extracted for ELISA-based NF-κB activity assay. Excess competitor oligonucleotides were coincubated with the nuclear protein of MIGRI-transfected and HSV-2-infected BCBL-1 cells for competition assays (HSV-2+MIGRI+Comp.). Results presented were the statistic of three independent experiments performed in triplicate. * *p*<0.05 and ** *p*<0.01 for Student's t-test versus Mock+MIGRI group, respectively; ^##^ *p*<0.01 and ^&^ *p*<0.01 for Student's t-test versus HSV-2+MIGRI group, respectively. (**B**). **Transfection of IKK_2_EE promoted HSV-2-induced activity of NF-κB in BCBL-1 cells.** BCBL-1 cells were infected by HSV-2 or Mock for 1 hour, and then were co-transfected with the plasmid IKK_2_EE and an NF-κB-dependent firefly luciferase reporter construct (0.4 µg) together with a Renilla luciferase construct pRL-TK (0.02 µg) for 12, 24 and 48 hours. Whole-cell extracts were prepared, and the firefly as well as the Renilla luciferase activity was measured. The experiment was performed three times in parallel, and the mean ± s.e.m is shown. ** *p*<0.01 and ^##^ *p*<0.01 for Student's t-test versus Mock+MIGRI and HSV-2+MIGRI groups, respectively. (**C**). **Transfection of IKK_2_EE inhibited ORF26 mRNA expression in HSV-2-infected BCBL-1 cells.** BCBL-1 cells were infected by HSV-2 or Mock for 1 hour, and then were transfected by IKK_2_EE or MIGRI for 12, 24 and 48 hours. Cells were collected and ORF26 mRNA expression in BCBL-1 cells was measured by RT-qPCR. Relative quantities of ORF26 expression represented on the y-axis. Results shown were from three independent experiments performed in triplicate. ** *p*<0.01 for Student's t-test versus Mock+MIGRI group; ^#^ *p*<0.05 and ^##^ *p*<0.01 for Student's t-test versus HSV-2+MIGRI group, respectively. (**D**). **Transfection of IKK_2_EE inhibited vIL-6 expression in HSV-2-infected BCBL-1 cells.** BCBL-1 cells were infected by HSV-2 or Mock for 1 hour, and then were transfected by IKK_2_EE or MIGRI for 12, 24 and 48 hours. Whole cell lysate were subjected to Western blot with the indicated antibody. (**E**). **Real-time DNA-PCR analysis for the viral copy number of KSHV.** BCBL-1 cells were infected by HSV-2 or Mock for 1 hour, and then were transfected by IKK_2_EE or MIGRI. At 24 and 48 hours postinfection, supernatant virus was collected, concentrated, and used for real-time DNA-PCR analysis. Results shown were from three independent experiments performed in triplicate. ** *p*<0.01 and ^##^ *p*<0.01 for Student's t-test versus Mock+MIGRI and HSV-2+MIGRI groups, respectively.

It has been shown that several KSHV genes and KSHV acute infection activated NF-κB signaling pathway in different cell lines, but the role of NF-κB during the reactivation of KSHV was still controversial [32]–[37]. To examine whether HSV-2 was able to trigger NF-κB activation in PEL cell lines, BCBL-1 cells were infected by HSV-2 and nuclear extracts were analyzed for NF-κB activation by ELISA-based NF-κB activity assay. As shown in Fig. 5A, HSV-2 infection of BCBL-1 cells increased phosphorylated p65 in nucleus to bind consensus binding sequences for p65 of NF-κB at 12, 24 and 48 hours, while wide-type p65 competitor oligo abolished this activation. To test the transcriptional function of activated NF-κB in response to HSV-2, we transfected an NF-κB-dependent firefly luciferase reporter construct into HSV-2-infected BCBL-1 cells and measured the NF-κB luciferase activity. We observed an apparent induction of reporter activity in BCBL-1 cells with HSV-2 infection compared to that of Mock-infected cells at 12, 24, and 48 hours (Fig. 5B). To define the function of NF-κB in HSV-2-induced KSHV reactivation, BCBL-1 cells were pre-treated by DMSO or Bay 11-7082, a pharmacologic inhibitor of IκB kinase(IKK)-NF-κB activation, for 1 hour followed by HSV-2 infection. It was demonstrated that treatment of Bay 11-7082 not only decreased the level of p65 in the nucleus (Fig. 5C), resulting in little amount of NF-κB binding to its DNA region in an NF-κB-dependent reporter construct (Fig. 5D), but also increased KSHV ORF26 mRNA and vIL-6 protein expression and viral particle release in HSV-2-infected BCBL-1 cells (Fig. 5E, 5F and 5G). On the other hand, transfection of IKK_2_EE, which was capable of phosphorylating IκBα, not only elevated the level of p65 in the nucleus and increased its function (Fig. 6A and 6B), but also down-regulated KSHV ORF26 mRNA and vIL-6 protein expression and viral particle release in HSV-2-infected BCBL-1 cells (Fig. 6C, 6D and 6E). Taken together, these data suggest that HSV-2 activated NF-κB pathway, which exhibit an inhibitory effect on KSHV reactivation in BCBL-1 cells.

### HIV-1 Tat Enhances Induction of KSHV Lytic Cycle Replication by HSV-2

**Figure 7:**
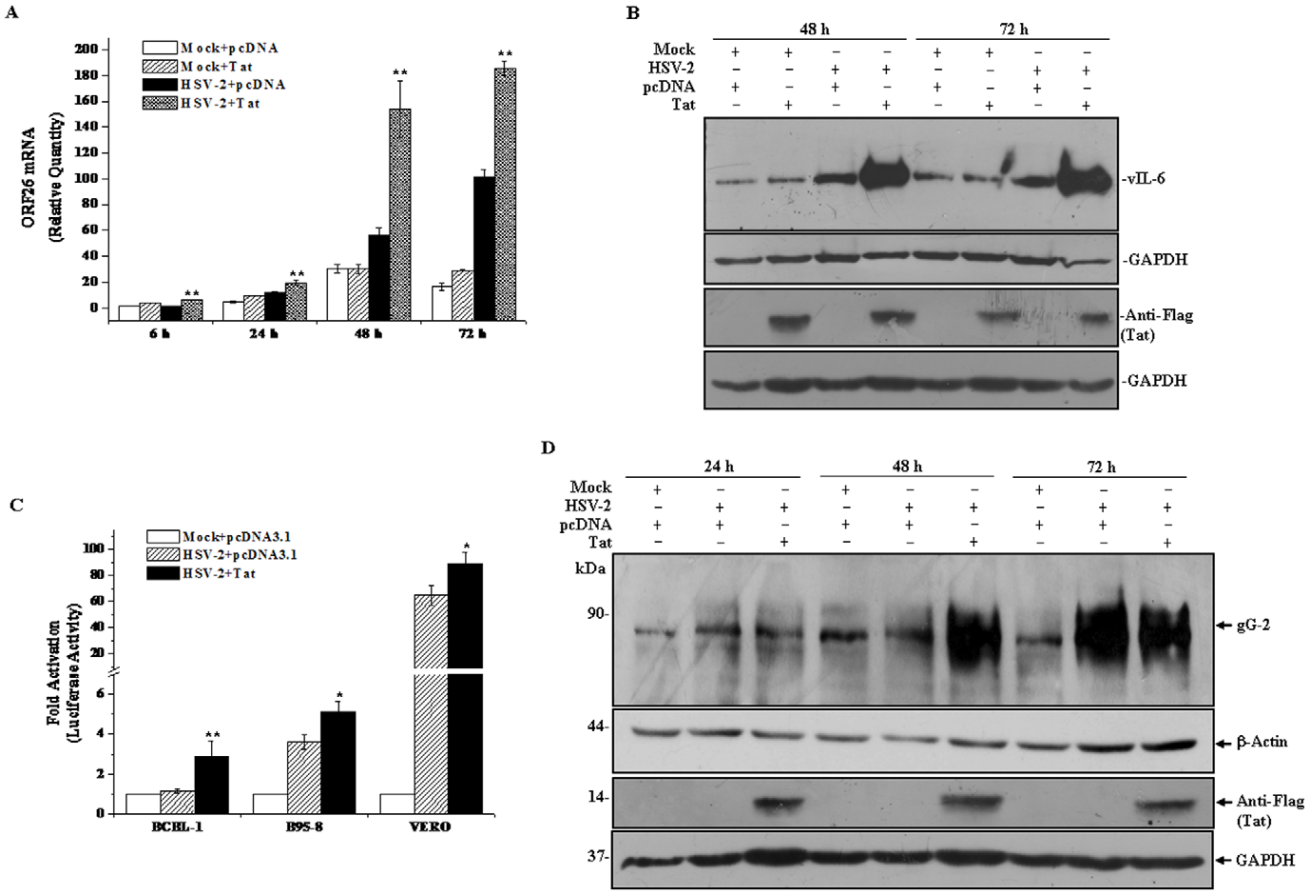
HIV-1 Tat cooperates with HSV-2 to induce KSHV lytic cycle replication. (**A**). **Effect of Tat on ORF26 mRNA expression in HSV-2-infected BCBL-1 cells.** Real-time quantitative PCR was performed to detect KSHV ORF26 mRNA expression in Mock-treated and pcDNA3.1-transfected BCBL-1 cells (**Mock+pcDNA**), Mock-treated and pTat-transfected BCBL-1 cells (**Mock+Tat**), or HSV-2-infected and pcDNA3.1-transfected BCBL-1 cells (**HSV-2+pcDNA**), HSV-2-infected and pTat-transfected BCBL-1 cells (**HSV-2+Tat**) for 6, 24, 48 and 72 hours. The results shown were from three independent experiments performed in triplicate. ** *p*<0.01 for Student's t-test versus HSV-2+pcDNA group. (**B**). **Effect of Tat on vIL-6 protein expression in HSV-2-infected BCBL-1 cells.** Cell lysates were collected from HSV-2-infected and pcDNA3.1-transfected BCBL-1 cells, or HSV-2-infected and pTat-transfected BCBL-1 cells for 48 and 72 hours. Whole cell lysate were subjected to Western blot with the indicated antibody. (**C**). **Effect of Tat on Rta promoter activities in HSV-2-infected BCBL-1 cells.** BCBL-1, B95-8 and Vero cells were infected with Mock and then co-transfected with pcDNA3.1, p50-Luc and Renilla vector pRL-TK (**Mock+pcDNA3.1**), infected with HSV-2 and then co-transfected with pcDNA3.1, p50-Luc and Renilla vector pRL-TK (**HSV-2+pcDNA3.1**), or infected with HSV-2 and then co-transfected with pTat, p50-Luc and Renilla vector pRL-TK (**HSV-2+Tat**). Luciferase activities were measured, normalized to Rellina inner control, and presented as fold increase (*n*-fold). All data points were the averages of three independent experiments performed in triplicate. * *p*<0.05 and ** *p*<0.01 for Student's t-test versus HSV-2+pcDNA3.1 group, respectively. (**D**). **Effect of Tat on HSV-2 replication in BCBL-1 cells.** BCBL-1 cells were infected with Mock and then transfected with pcDNA3.1 (**Mock+pcDNA**), infected with HSV-2 and then transfected with pcDNA3.1 (**HSV-2+pcDNA**), or infected with HSV-2 and then transfected with pTat (**HSV-2+pTat**) for 24, 48 and 72 hours. Whole cell lysate were subjected to Western blot with the indicated antibody.

To explore whether Tat influenced HSV-2-induced KSHV replication, BCBL-1 cells were infected with HSV-2 and then transfected with pTat. RT-qPCR was performed to detect KSHV ORF26 mRNA expression. ORF26 mRNA expression in HSV-2-infected and Tat-transfected BCBL-1 cells increased 3.03-fold at 6 hours, 1.94-fold at 24 hours, 2.95-fold at 48 hours and 1.64-fold at 72 hours, respectively, when compared to that of HSV-2-infected and pcDNA-transfected BCBL-1 cells (Fig. 7A). These data demonstrated that intracellular Tat enhanced ORF26 mRNA expression in HSV-2-induced KSHV lytic cycle phase. Interestingly, the expression of ORF26 was slightly up-regulated in Tat-transfected and mock-treated BCBL-1 cells at 6, 24, and 72 hours compared with that of pcDNA-transfected and mock-treated BCBL-1 cells. However, our previous report showed that intracellular Tat did not influence the expression of ORF26 mRNA in BCBL-1 cells until at 96 hours [12]. It was probably that in this study the supernatant of Vero cell cultures contained some soluble factors, which might have a synergetic effect with Tat on KSHV reactivation. To investigate whether Tat has an effect on the protein level of vIL-6, Western blot analysis was performed. As shown in Fig. 7B, HSV-2-infected BCBL-1 cells, which were transfected with pTat, expressed more vIL-6 at 48 and 72 hours, compared with HSV-2-infected BCBL-1 cells transfected with pcDNA. These data indicated that the Tat might enhance HSV-2-induced KSHV replication.

To explore whether Tat increased HSV-2-induced KSHV replication via activating Rta, we examined the effect of Tat on Rta promoter activity in HSV-2-infected BCBL-1, B95-8 and Vero cells. Rta promoter activity in pTat and p50-Luc-cotransfected BCBL-1 cells infected by HSV-2 increased 2.48-fold when compared to that of pcDNA and p50-Luc-cotransfected BCBL-1 cells infected by HSV-2. Similar expression pattern of Rta promoter activity was also observed in B95-8 and Vero cell (Fig. 7C). Taken these results together, we speculated that Tat enhanced the HSV-2-induced KSHV reactivation in BCBL-1 cells by activating Rta promoter.

Because HSV-2-infected BCBL-1 cells expressed type specific glycoprotein gG of HSV-2 (Fig. 1C), we next tested the effect of Tat on HSV-2 gG expression in BCBL-1 cells. As shown in Fig. 7D, HSV-2-infected BCBL-1 cells consistently expressed HSV-2 gG at 72 hours, no matter transfected with pcDNA or pTat, which was consistent with the results of Fig. 1C at 48 hours. Remarkably, at 48 hours, gG was detectable in HSV-2-infected and pTat-transfected BCBL-1 cells while there was no corresponding band detected in HSV-2-infected and pcDNA-transfected BCBL-1 cells (Fig. 7D), implying that overexpression of Tat promotes HSV-2 replication. Together these data suggest that Tat might enhance KSHV lytic cycle replication by accelerating HSV-2 replication in BCBL-1 cells.

## Discussion

The epidemical studies shown that interaction between the STIs was complex. The relationship of HSV-2 and KSHV remains quite controversial. Studies from three groups demonstrated that HSV-2 and KSHV were associated in the prisoners of Italian and some areas in Africa, which suggest that HSV-2 infection contributed to sexual transmission of KSHV infection [18]–[20]. However, Malope et al have shown that in a heterosexual South African population there was no evidence of sexual transmission of KSHV [38].

The present work demonstrated that HSV-2 infection of BCBL-1 cells induced KSHV replication and provided the direct laboratory evidence that HSV-2 was a potential cofactor for KSHV reactivation. KSHV was necessary but not sufficient in KS development and the cofactors played an important role in the pathogenesis of KS [8]. For instance, HIV-1 infection of PEL cell lines induced the reactivation of KSHV through directly activating the promoter of KSHV Rta [13], [39], [40]. HSV-1 could activate KSHV replication partially by elevating the level of IL-10 and IL-4 [11]. Here we have further demonstrated that HSV-2 was also a critical factor responsible for the induction of KSHV replication, suggesting that HSV-2 might promote KS progression by increasing viral load. However, whether some soluble factors produced in response to HSV-2 infection might also be involved in this procedure is still unknown.

The role of NF-κB pathway during the reactivation of KSHV is still a highly controversial and arguable subject. We and other five groups consistently demonstrated that NF-κB signaling displayed an inhibitory effect on KSHV reactivation [33], [36], [37], [41]–[43]. However, one group showed that activation of NF-κB was required for KSHV replication and the production of replication-competent KSHV virions [32]. Actually, Grossmann and colleagues indicated that the relationship between NF-κB and spontaneous KSHV reactivation was complex, non-uniform and dependent on the cellular context. Even though NF-κB activation is inhibitory to lytic gene expression in some contexts, such inhibition is at least partially bypassed or overridden during lytic growth. In this study, we indicated that HSV-2 infection of BCBL-1 cells led to dramatic activation of the NF-κB signaling, which was consistent with previous reports [44], [45]. Furthermore, inhibition of NF-κB significantly promoted KSHV lytic replication; elevating NF-κB signaling remarkably repressed KSHV replication, which was in agreement with the reports of six groups above mentioned. These observations also implied that other signal pathways might exist together with activated NF-κB in HSV-2-infected BCBL-1 cells during KSHV reactivation. When HSV-2 infected BCBL-1 cells, it must lead to alternation of multiple signal pathways. Some activated signals facilitated KSHV replication; inversely, others exerted an inhibitory effect, such as NF-κB. If signals whose activation can promote KSHV replication are always predominated, they may compensate the inhibitory effect of NF-κB. Therefore, our results never eliminate the possibility that some other signals produced in response to HSV-2 infection might also be involved in this procedure.

The Tat protein of HIV-1 functioned as a transacting transcriptional activator and was of great interest in the studies of KS. Our previous studies demonstrated that Tat not only induced KSHV replication, but also accelerated the tumorigenesis by KSHV Kaposin A *in vitro* and *in vivo* [12], [46]. In the current study, we showed that Tat could enhance the HSV-2-induced lytic replication of KSHV. Interestingly, Tat also accelerated HSV-2 replication in BCBL-1 cells. Given the fact that HSV-1 and its immediate-early genes ICP0 and ICP4 greatly stimulated the expression of HIV-1 LTR-directed viral gene expression collaborated with Tat [47], [48], and Tat increased the transcriptional activity of HSV-1 ICP0 [48]. We speculated that, with high homology to ICP0 and ICP4 of HSV-1 [49], the cooperation of Tat and ICP0 and ICP4 of HSV-2 stimulated the transcript and replication of HSV-2, which indirectly induced KSHV replication. However, the mechanism still needs to be elucidated.

In summary, we showed that HSV-2 was a potentially important factor in the pathogenesis of KS and NF-κB signal exerted an inhibitory effect in HSV-2-induced KSHV replication. We also revealed that Tat could enhance HSV-2-induced KSHV replication. Because HSV-2 infection of cells stimulated multiple downstream effects [50], [51] and intracellular Tat activated the KSHV replication partially through JAK/STAT pathways [12], further studies are needed to better understand the signaling involved in Tat increasing HSV-2-induced KSHV reactivation from latency.

## Materials and Methods

### Cell Culture and Virus Infection

The BCBL-1 and BC-3 cells (EBV-negative and KSHV-positive PEL cell lines) were obtained through the AIDS Research and Reference Reagent Program, National Institutes of Health. A HEK293 cell line that harbors the KSHV genome inserted into a bacterial artificial chromosome, designated 293/Bac36, was a kind gift from S-J Gao (University of Southern California, Los Angeles, CA). B95-8, which were KSHV-negative and EBV-positive marmoset B-cell lines, HEK293, and Vero (African green monkey kidney fibroblasts) cells were obtained from American Type Culture Collection (ATCC, Rockville, MD). Both BCBL-1 and B95-8 cells were maintained in RPMI-1640 containing 10% heat-inactivated fetal bovine serum (FBS), 2 mmol/L L-glutamine, 100 U/ml penicillin, and 100 µg/ml streptomycin. HEK293 and Vero cells were maintained in DMEM+10% FBS. 293/Bac36 was grown under the same conditions, except with the addition of 100 mg/mL hygromycin B. HSV-2 (333 strain) propagated in Vero cells as previously described [11], [52]. The supernatants from normal Vero cells culture were collected and used as a control (Mock) to treat target cells.

### Antibodies and Reagents

Anti-HSV-2 Glycoprotein G-2 (gG-2) mouse monoclonal antibody (MAb) was purchased from Biodesign International™. Anti-KSHV ORF59 MAb and viral IL-6 (vIL-6) rabbit polyclonal antibody (PAb) were obtained from Advanced Biotechnologies Inc. (Columbia, MD). Anti-KSHV Rta (also known as ORF50) peptide antibodies were generated by immunization of rabbits with a Rta peptides (aa667–691). The horseradish peroxidase (HRP)-conjugated donkey anti-goat and goat anti-mouse and -rabbit IgG, and mouse MAb to β-Actin, α-Tubulin and GAPDH (used to monitor sample loading) were purchased from Santa Cruz Biotechnologies (Santa Cruz, Calif.). Anti-Flag M2 mouse MAb were obtained from Cell Signaling Technologies (Beverly, MA, USA). Bay11-7082 were purchased from Sigma (St. Louis, MO, USA).

### Western Blot Analysis

Western blot was performed as described previously [12]. Proteins were visualized with ECL reagents (Amersham Biosciences, Piscataway, NJ) according to the manufacturer's instructions. Western blot experiments were repeated at least three times to confirm results.

### Real-Time Quantitative Reverse Transcription-PCR (RT-qPCR)

RT-qPCR was performed in the 7300 Real-Time PCR System (Applied Biosystems, Foster City, CA). The sequences of KSHV-specific primers and probes including Rta and 26 were shown as previously described [11].

### Real-time DNA-PCR Analysis for Viral Copy Number

Real-time DNA-PCR was carried out on viral DNA from supernatants of BCBL-1 cells essentially as described previously [53]. Specifically, the primers and probe for the real-time DNA-PCR were designed to detect ORF26 [12]. Viral DNA was extracted using the high pure viral nucleic acid kit (Roche, Germany) as per the manufacturer's instructions. The KSHV ORF26 gene cloned in the pcDNA3.1 (Invitrogen, Inc., Carlsbad, CA) was used to generate the standard curve.

### RT-PCR

RT-PCR was performed as previously described [11]. Primers used for analysis of KSHV genes in this study were also shown as previously described [11]. Primers used for analysis of HSV-2 genes are listed in Table 1.

**Table 1:** Sets of primers of HSV-2 used for RT-PCR.

mRNA	Oligonucleotides	Accession no.	Expected size (bp)	Annealingtemp (°C)	Cycles
UL48	F:5′-GAG CAG GCC CTC GAT TGT C-3′ R:5′-CCA GTT GGC GTG TCT GTT TCA-3′	NC-001798.1	352	58	29
UL5	F:5′-GTG ATG CGA CTG GCG TTG G-3′ R:5′-CAG TTT GTG GAC CGC TTT GT-3′	NC-001798.1	278	58	30
US6	F:5′-GCG TGT TTA CCA CAT TCA GCC-3′ R:5′-TCC GTC CAG TCG TTT ATC TTC A-3′	NC-001798.1	420	58	29
β-Actin	F:5′-TGA CGG GGT CAC CCA CAC TGT GCC CAT CTA-3′ R:5′-CTA GAA GCA TTT GCG GTG GAC GAT GGA GGG-3′	BC016045	661	56	24

The oligonucleotides were selected from the sequences with the indicated accession number. The size of each amplified product, its annealing temperature, and numbers of PCR cycles are indicated. F, forward; R, reverse.

### Immunofluorescence Assay (IFA)

IFA was performed as described elsewhere. Briefly, after infection of BCBL-1 cells with HSV-2, cells (10^7^/ml) were washed and smeared on chamber slides. Slides were incubated with a 1∶100 dilution of anti-KSHV ORF59 mouse MAb. Alexa Fluor 568 (Invitrogen)-conjugated goat anti-mouse antibody (1∶200 dilution) was used as a secondary antibody for detection. The cells were counterstained with 4′,′-diamidino-2-phenylindole. Images were observed and recorded with a Zeiss Axiovert 200 M epifluorescence microscope (Carl Zeiss, Inc.). Photographs of at least 10 unique fields were taken of every slide, and the number of positive and negative cells was counted separately by three individuals, including one who was blinded to the results to calculate the percentage of positive cells. Immunostaining was performed on samples from three separate experiments.

### Plasmid, Transfection and Luciferase Reporter Assay

The pTat plasmid was used as described previously [12]. The siRNA construct (pRNAT-Rta) that targets KSHV Rta was utilized as previously described [11]. NF-κB reporter plasmid containing three tandem repeats of consensus NF-κB binding sites was obtained from Huaguo Xu (Department of Clinical Laboratory, Nanjing Medical University, Nanjing, China). IKK_2_EE capable of phosphorylating IκBα and control vector MIGRI were kindly provided by Zan Huang (Wuhan University School of Life Science, Wuhan, China). The KSHV Rta luciferase reporter construct (p50-Luc) was generated as described previously [12]. Before transfection, cells were adsorbed with HSV-2 for 1 hour. After 48-hour transfection, cells were harvested for luciferase assay. Renilla vector pRL-TK (Promega) was used as an internal control and relative luciferase activity was assayed using the Promega dual-luciferase reporter assay system. BCBL-1, BC-3 and B95-8 cells were electroporated at 250 V and 960 microfarads using a Gene Pulser (Bio-Rad Laboratories, Hercules, CA) as described previously [43]. Both 293/Bac36 and Vero cells were transfected with Lipofectamine 2000 (Invitrogen) following the manufacturer's instructions.

### ELISA-based Transcription Factor Activity Assay

The activation of the transcription factor NF-κB was assayed by using TransFactor NF-κB p65 Colorimetric Kit from Clontech Laboratories, Inc (California, USA) under conditions recommended by the manufacturer and as described elsewhere [54]. Nuclear protein of BCBL-1 cells was extracted using the Nuclear Extract Kit (Active Motif, CA, USA) and ELISA-based transcription factor activity assay was performed following the manufacturer's protocol. For competition assays, competitor oligonucleotides were added to coincubate with nuclear protein. Samples were run in triplicates, and all experiments were performed on three occasions.
